# Supplementary figures and images for: Hypoxaemia prevalence and management among children and adults presenting to primary care facilities in Uganda: A prospective cohort study
Source: PLOS Glob Public Health. 2022 Apr 22;2(4):e0000352. doi: 10.1371/journal.pgph.0000352 (PMC10022140; doi:10.1371/journal.pgph.0000352)

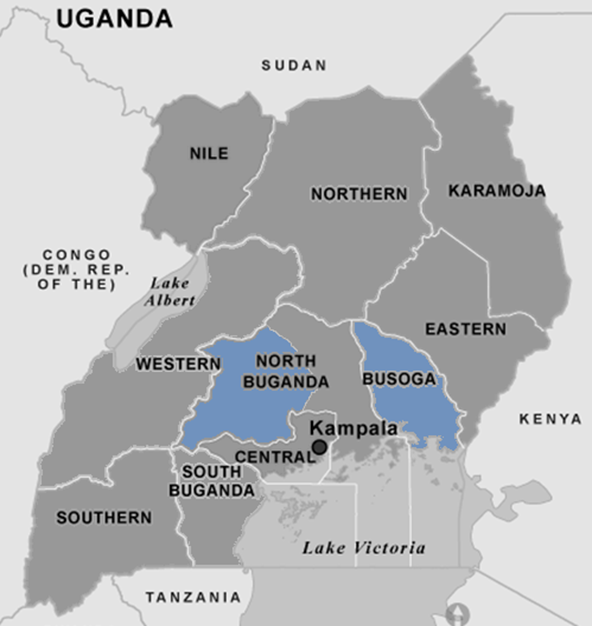

Supplement: S1 Fig — Blue shaded areas indicating the approximate catchment areas of Mubende and Jinja Regional referral hospitals. (TIF) [file pgph.0000352.s001.tif]

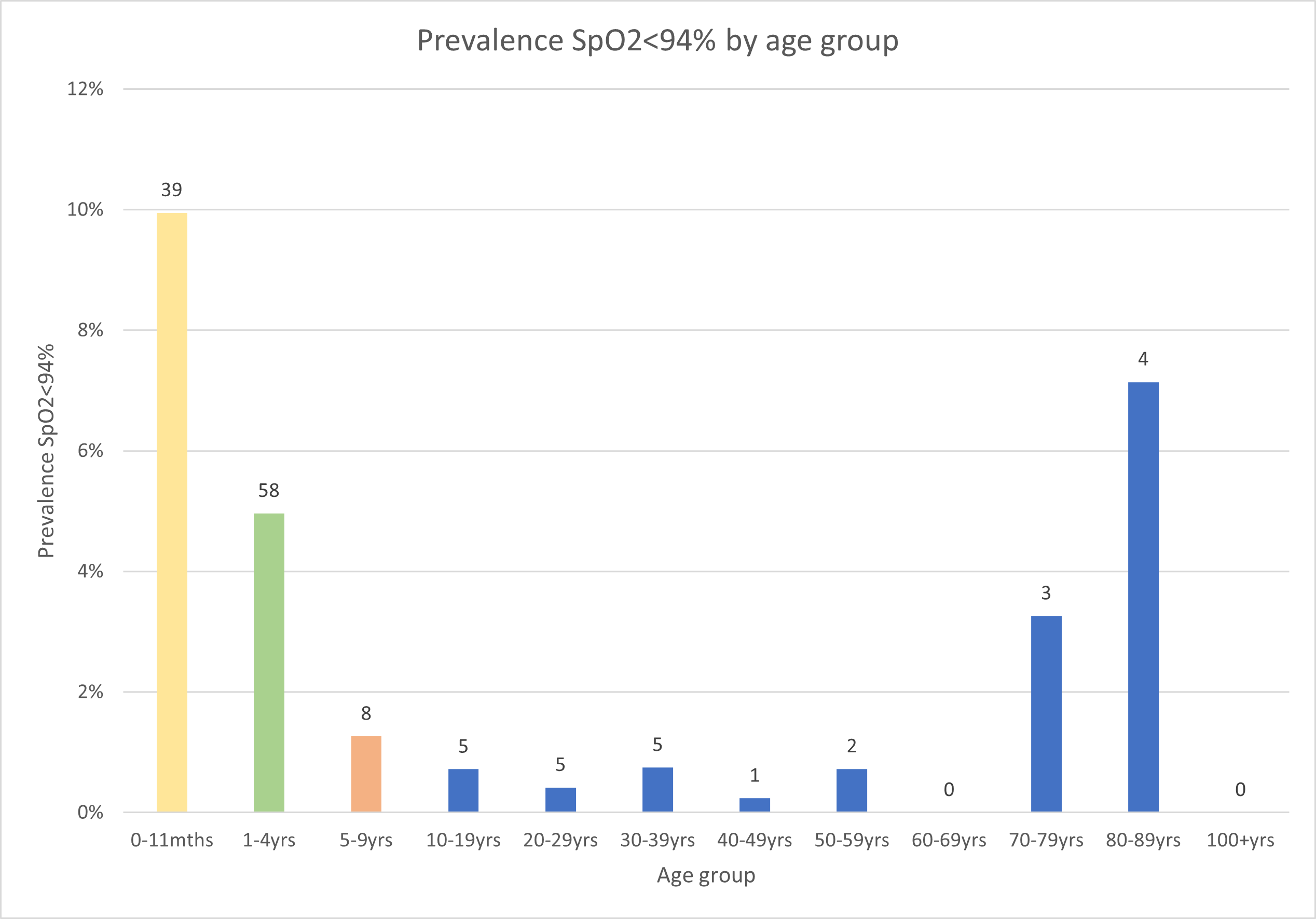

Supplement: S2 Fig — Number labels on each bar represent the number of hypoxaemia cases. (TIF) [file pgph.0000352.s002.tif]
