# Supplementary material for: Hypoxaemia prevalence and management among children and adults presenting to primary care facilities in Uganda: A prospective cohort study
Source: PLOS Glob Public Health. 2022 Apr 22;2(4):e0000352. doi: 10.1371/journal.pgph.0000352 (PMC10022140; doi:10.1371/journal.pgph.0000352)
Supplement: S1 Table — Presenting complaints as reported by patient/caregiver. Diagnosis as recorded by treating healthcare worker. P-values obtained from Pearson’s chi-squared or Fishers exact test as applicable. ARI = acute respiratory infection; STI–sexually transmitted infection; UTI–urinary tract infection. (DOCX) [file pgph.0000352.s003.docx]

## S1 TABLE: Demographic and clinical features of 5780 acutely unwell children, adolescents, and adults presenting to primary care (HCIII) facilities in Uganda, Feb-Apr 2021

| **DEMOGRAPHIC INFORMATION** | | | | | | |  |
| --- | --- | --- | --- | --- | --- | --- | --- |
| **Age group** | Busoga | | North Central | | Total | |  |
| Neonate | 10 | 0.2% | 6 | 0.4% | 16 | 0.3% |  |
| 1-11 mths | 280 | 6.8% | 96 | 5.8% | 376 | 6.5% |  |
| 1-4 yrs | 911 | 22.1% | *258* | 15.5% | 1169 | 20.2% |  |
| 5-9yrs | 469 | 11.4% | *164* | 9.9% | 633 | 11.0% |  |
| 10-14 yrs | 202 | 4.9% | *100* | 6.0% | 302 | 5.2% |  |
| 15-24 yrs | 793 | 19.2% | *295* | 17.8% | 1088 | 18.8% |  |
| 25-49 yrs | 1,072 | 26.0% | 530 | 31.9% | 1602 | 27.7% |  |
| 50+ yrs | 383 | 9.3% | 211 | 12.7% | 594 | 10.3% |  |
| *Total* | *4120* |  | *1660* |  | *5780* |  |  |
| *<5 years* | 1201 | 29.2% | 360 | 21.7% | *1561* | 27.0% |  |
| *5-14 yrs* | 671 | 16.3% | 264 | 15.9% | *935* | 16.2% |  |
| *>= 15yrs* | 2248 | 54.6% | 1036 | 62.4% | *3284* | 56.8% | *P<0.001* |
| **Sex** | Busoga | | North Central | | Total | |  |
| Male | 1207 | 29.3% | 654 | 39.4% | 1861 | 32.2% |  |
| Female | 2913 | 70.7% | 1006 | 60.6% | 3919 | 67.8% | *P<0.001* |
| **Vital signs documented** | Busoga | | North Central | | Total | |  |
| Temperature | 303 | 7.4% | 159 | 9.6% | 462 | 8.0% | *P=0.005* |
| Heart rate | 2867 | 69.6% | 1036 | 62.4% | 3903 | 67.5% | *P<0.001* |
| Respiratory rate | 12 | 0.3% | 2 | 0.1% | 14 | 0.2% | *P=0.232* |
| Blood pressure | 98 | 2.4% | 57 | 3.4% | 155 | 2.7% | *P=0.024* |
| **PRESENTING COMPLAINTS** | | | | | | |  |
| **Fever/chills** | Busoga | | North Central | | Total | |  |
| *<5 yrs* | 1,002 | 83.4% | 206 | 57.2% | 1208 | 77.4% |  |
| *5-14 yrs* | 481 | 71.7% | 146 | 55.3% | 627 | 67.1% |  |
| >=15 yrs | 871 | 38.7% | 459 | 44.3% | 1330 | 40.5% |  |
| *Total* | *2354* | *57.1%* | *811* | *48.9%* | *3165* | *54.8%* |  |
| **Respiratory complaints** | Busoga | | North Central | | Total |  |  |
| *<5 yrs* | 885 | 73.7% | 273 | 75.8% | 1158 | 74.2% |  |
| *5-14 yrs* | 367 | 54.7% | 144 | 54.5% | 511 | 54.7% |  |
| >=15 yrs | 800 | 35.6% | 400 | 38.6% | 1200 | 36.5% |  |
| *Total* | *2052* | *49.8%* | *817* | *49.2%* | *2869* | *49.6%* |  |
| **Fever/chills OR Respiratory** | Busoga | | North Central | | Total | |  |
| *<5 yrs* | 1,148 | 95.6% | 330 | 91.7% | 1478 | 94.7% |  |
| *5-14 yrs* | 585 | 87.2% | 208 | 78.8% | 793 | 84.8% |  |
| >=15 yrs | 1329 | 59.1% | 686 | 66.2% | 2015 | 61.4% |  |
| *Total* | *3062* | *74.3%* | *1224* | *73.7%* | *4286* | *74.2%* |  |
| **Pain** | Busoga | | North Central | | Total | |  |
| *<5 yrs* | 147 | 12.2% | 37 | 10.3% | 184 | 11.8% |  |
| *5-14 yrs* | 359 | 53.5% | 89 | 33.7% | 448 | 47.9% |  |
| >=15 yrs | 1611 | 71.7% | 554 | 53.5% | 2165 | 65.9% |  |
| *Total* | *2117* | *51.4%* | *680* | *41.0%* | *2797* | *48.4%* |  |
| **Abdominal complaints** | Busoga | | North Central | | Total | |  |
| *<5 yrs* | 233 | 19.4% | 39 | 10.8% | 272 | 17.4% |  |
| *5-14 yrs* | 258 | 38.5% | 77 | 29.2% | 335 | 35.8% |  |
| *>= 15yrs* | 905 | 40.3% | 344 | 33.2% | 1249 | 38.0% |  |
| *Total* | *1396* | *33.9%* | *460* | *27.7%* | *1856* | *32.1%* |  |
| **Diarrhoea or vomiting** | Busoga | | North Central | | Total | |  |
| *<5 yrs* | 362 | 30.1% | 97 | 26.9% | 459 | 29.4% |  |
| *5-14 yrs* | 131 | 19.5% | 41 | 15.5% | 172 | 18.4% |  |
| >=15 yrs | 153 | 6.8% | 79 | 7.6% | 232 | 7.1% |  |
| *Total* | *646* | *15.7%* | *217* | *13.1%* | *863* | *14.9%* |  |
| **Urogenital complaints** | Busoga | | North Central | | Total | |  |
| *<5 yrs* | 10 | 0.8% | 5 | 1.4% | 15 | 1.0% |  |
| *5-14 yrs* | 15 | 2.2% | 7 | 2.7% | 22 | 2.4% |  |
| >=15 yrs | 208 | 9.3% | 103 | 9.9% | 311 | 9.5% |  |
| *Total* | *233* | *5.7%* | *115* | *6.9%* | *348* | *6.0%* |  |
| **Skin complaints** | Busoga | | North Central | | Total | |  |
| *<5 yrs* | 54 | 4.5% | 21 | 5.8% | 75 | 4.8% |  |
| *5-14 yrs* | 21 | 3.1% | 11 | 4.2% | 32 | 3.4% |  |
| >=15 yrs | 39 | 1.7% | 30 | 2.9% | 69 | 2.1% |  |
| *Total* | *114* | *2.8%* | *62* | *3.7%* | *176* | *3.0%* |  |
| **Ear, eye, dental complaints** | Busoga | | North Central | | Total | |  |
| *<5 yrs* | 27 | 2.2% | 25 | 6.9% | 52 | 3.3% |  |
| *5-14 yrs* | 17 | 2.5% | 18 | 6.8% | 35 | 3.7% |  |
| >=15 yrs | 48 | 2.1% | 34 | 3.3% | 82 | 2.5% |  |
| *Total* | *92* | *2.2%* | *77* | *4.6%* | *169* | *2.9%* |  |
| **All other complaints** | Busoga | | North Central | | Total | |  |
| *<5 yrs* | 277 | 23.1% | 51 | 14.2% | 328 | 21.0% |  |
| *5-14 yrs* | 137 | 20.4% | 38 | 14.4% | 175 | 18.7% |  |
| >=15 yrs | 623 | 27.7% | 207 | 20.0% | 830 | 25.3% |  |
| *Total* | *1037* | *25.2%* | *296* | *17.8%* | *1333* | *23.1%* |  |
| **Other complaint as only complaint** | Busoga | | North Central | | Total | |  |
| *<5 yrs* | 8 | 0.7% | 6 | 1.7% | 14 | 0.9% |  |
| *5-14 yrs* | 2 | 0.3% | 6 | 2.3% | 8 | 0.9% |  |
| >=15 yrs | 34 | 1.5% | 29 | 2.8% | 63 | 1.9% |  |
| *Total* | *44* | *1.1%* | *41* | *2.5%* | *85* | *1.5%* |  |
| **DIAGNOSES** | | | | | | |  |
| **Malaria** | Busoga | | North Central | | Total | |  |
| *<5 yrs* | 751 | 62.5% | 74 | 20.6% | 825 | 52.9% |  |
| *5-14 yrs* | 461 | 68.7% | 77 | 29.2% | 538 | 57.5% |  |
| >=15 yrs | 799 | 35.5% | 166 | 16.0% | 965 | 29.4% |  |
| *Total* | *2011* | *48.8%* | *317* | *19.1%* | *2328* | *40.3%* |  |
| **ARI** | Busoga | | North Central | | Total | |  |
| *<5 yrs* | 570 | 47.5% | 241 | 66.9% | 811 | 52.0% |  |
| *5-14 yrs* | 242 | 36.1% | 121 | 45.8% | 363 | 38.8% |  |
| >=15 yrs | 648 | 28.8% | 356 | 34.4% | 1004 | 30.6% |  |
| *Total* | *1460* | *35.4%* | *718* | *43.3%* | *2178* | *37.7%* |  |
| **Diarrhoeal disease** | Busoga | | North Central | | Total | |  |
| *<5 yrs* | 178 | 14.8% | 65 | 18.1% | 243 | 15.6% |  |
| *5-14 yrs* | 39 | 5.8% | 18 | 6.8% | 57 | 6.1% |  |
| >=15 yrs | 84 | 3.7% | 29 | 2.8% | 113 | 3.4% |  |
| *Total* | *301* | *7.3%* | *112* | *6.7%* | *413* | *7.1%* |  |
| **Helminths** | Busoga | | North Central | | Total | |  |
| *<5 yrs* | 27 | 2.2% | 14 | 3.9% | 41 | 2.6% |  |
| *5-14 yrs* | 28 | 4.2% | 18 | 6.8% | 46 | 4.9% |  |
| >=15 yrs | 46 | 2.0% | 20 | 1.9% | 66 | 2.0% |  |
| *Total* | *101* | *2.5%* | *52* | *3.1%* | *153* | *2.6%* |  |
| **Skin disorders** | Busoga | | North Central | | Total | |  |
| *<5 yrs* | 43 | 3.6% | 26 | 7.2% | 69 | 4.4% |  |
| *5-14 yrs* | 16 | 2.4% | 11 | 4.2% | 27 | 2.9% |  |
| >=15 yrs | 43 | 1.9% | 20 | 1.9% | 63 | 1.9% |  |
| *Total* | *102* | *2.5%* | *57* | *3.4%* | *159* | *2.8%* |  |
| **STI/Genital/UTI** | Busoga | | North Central | | Total | |  |
| *<5 yrs* | 4 | 0.3% | 3 | 0.8% | 7 | 0.4% |  |
| *5-14 yrs* | 10 | 1.5% | 10 | 3.8% | 20 | 2.1% |  |
| >=15 yrs | 404 | 18.0% | 216 | 20.8% | 620 | 18.9% |  |
| *Total* | *418* | *10.1%* | *229* | *13.8%* | *647* | *11.2%* |  |
| **Septicaemia** | Busoga | | North Central | | Total | |  |
| *<5 yrs* | 34 | 2.8% | 7 | 1.9% | 41 | 2.6% |  |
| *5-14 yrs* | 9 | 1.3% | 5 | 1.9% | 14 | 1.5% |  |
| >=15 yrs | 36 | 1.6% | 20 | 1.9% | 56 | 1.7% |  |
| *Total* | *79* | *1.9%* | *32* | *1.9%* | *111* | *1.9%* |  |
| **Non-diarrhoeal digestive system illnesses** | Busoga | | North Central | | Total | |  |
| *<5 yrs* | 6 | 0.5% | 2 | 0.6% | 8 | 0.5% |  |
| *5-14 yrs* | 10 | 1.5% | 6 | 2.3% | 16 | 1.7% |  |
| >=15 yrs | 280 | 12.5% | 95 | 9.2% | 375 | 11.4% |  |
| *Total* | *296* | *7.2%* | *103* | *6.2%* | *399* | *6.9%* |  |

Presenting complaints as reported by patient/caregiver. Diagnosis as recorded by treating healthcare worker. P-values obtained from Pearson’s chi-squared or Fishers exact test as applicable. ARI = acute respiratory infection; STI – sexually transmitted infection; UTI – urinary tract infection.
