# Supplementary material for: Hypoxaemia prevalence and management among children and adults presenting to primary care facilities in Uganda: A prospective cohort study
Source: PLOS Glob Public Health. 2022 Apr 22;2(4):e0000352. doi: 10.1371/journal.pgph.0000352 (PMC10022140; doi:10.1371/journal.pgph.0000352)
Supplement: S2 Table — ARI = acute respiratory infection; CI = confidence interval; Dx = diagnoses; GIT = gastrointestinal tract; STI = sexually transmitted infection; UTI = urinary tract infection. (DOCX) [file pgph.0000352.s004.docx]

## **S2 TABLE** Prevalence of hypoxaemia among acutely unwell children, adolescents and adults presenting to HCIII facilities in Uganda, Feb-Apr 2021 (extended results)

|  | **N** | **%** | **Severe hypoxaemia (SpO_2_<90%)** | | | | | **Moderate hypoxaemia (SpO_2_ 90-93%)** | | | | |
| --- | --- | --- | --- | --- | --- | --- | --- | --- | --- | --- | --- | --- |
|  |  |  | **N** | **%** | **prevalence** | **95% CI** | | **N** | **%** | **prevalence** | **95% CI** | |
| **Neonate** | 16 | 0.3% | 0 | . | . | . | . | 4 | 3.9% | 25.0% | 8.9% | 53.3% |
| **1-11 months** | 376 | 6.5% | 8 | 29.6% | 2.1% | 1.1% | 4.2% | 27 | 26.2% | 7.2% | 5.0% | 10.3% |
| **1-4 years** | 1,169 | 20.2% | 13 | 48.1% | 1.1% | 0.6% | 1.9% | 45 | 43.7% | 3.8% | 2.9% | 5.1% |
| **5-9 years** | 633 | 11.0% | 3 | 11.1% | 0.5% | 0.2% | 1.5% | 5 | 4.9% | 0.8% | 0.3% | 1.9% |
| **10-14 years** | 302 | 5.2% | 0 | . | . | . | . | 5 | 4.9% | 1.7% | 0.7% | 3.9% |
| **15-24 years** | 1,088 | 18.8% | 0 | . | . | . | . | 3 | 2.9% | 0.3% | 0.1% | 0.9% |
| **25-49 years** | 1,602 | 27.7% | 1 | 3.7% | 0.1% | 0.0% | 0.4% | 7 | 6.8% | 0.4% | 0.2% | 0.9% |
| **50+ years** | 594 | 10.3% | 2 | 7.4% | 0.3% | 0.1% | 1.3% | 7 | 6.8% | 1.2% | 0.6% | 2.5% |
| ***Total*** | **5780** | **100%** | **27** | **100%** | **0.5%** | **0.3%** | **0.7%** | **103** | **100%** | **1.8%** | **1.5%** | **2.2%** |
|  |  |  |  |  |  |  |  |  |  |  |  |  |
| **Girls (<5 years)** | 790 | 13.7% | 14 | 51.9% | 1.8% | 1.1% | 3.0% | 38 | 36.9% | 4.8% | 3.5% | 6.5% |
| **Boys (<5 years)** | 771 | 13.3% | 7 | 25.9% | 0.9% | 0.4% | 1.9% | 38 | 36.9% | 4.9% | 3.6% | 6.7% |
| **Girls (5-14 years)** | 568 | 9.8% | 1 | 3.7% | 0.2% | 0.0% | 1.2% | 4 | 3.9% | 0.7% | 0.3% | 1.9% |
| **Boys (5-14 years)** | 367 | 6.3% | 2 | 7.4% | 0.5% | 0.1% | 2.2% | 6 | 5.8% | 1.6% | 0.7% | 3.6% |
| **Women (≥15 years)** | 2,561 | 44.3% | 1 | 3.7% | 0.0% | 0.0% | 0.3% | 14 | 13.6% | 0.5% | 0.3% | 0.9% |
| **Men (≥15 years)** | 723 | 12.5% | 2 | 7.4% | 0.3% | 0.1% | 1.1% | 3 | 2.9% | 0.4% | 0.1% | 1.3% |
| ***Total*** | **5,780** | **100%** | **27** | **100%** | **0.5%** | **0.3%** | **0.7%** | **103** | **100%** | **1.8%** | **1.5%** | **2.2%** |
|  |  |  |  |  |  |  |  |  |  |  |  |  |
| **Busoga** | 4,120 | 71.3% | 20 | 74.1% | 0.5% | 0.3% | 0.7% | 86 | 83.5% | 2.1% | 1.7% | 2.6% |
| **North Central** | 1,660 | 28.7% | 7 | 25.9% | 0.4% | 0.2% | 0.9% | 17 | 16.5% | 1.0% | 0.6% | 1.6% |
| ***Total*** | **5,780** | **100%** | **27** | **100%** | **0.5%** | **0.3%** | **0.7%** | **103** | **100%** | **1.8%** | **1.5%** | **2.2%** |

| **HYPOXAEMIA PREVALENCE BY PRESENTING COMPLAINT** | | | | | | | | | | | | |
| --- | --- | --- | --- | --- | --- | --- | --- | --- | --- | --- | --- | --- |
|  | **N** | **%** | **Severe hypoxaemia (SpO_2_<90%)** | | | | | **Moderate hypoxaemia (SpO_2_ 90-93%)** | | | | |
|  |  |  | **N** | **%** | **prevalence** | **95% CI** | | **N** | **%** | **prevalence** | **95% CI** | |
| **U5 years by presenting complaints** | | | | | | | | | | | | |
| **Abdominal** | 272 | 17.4% | 2 | 9.5% | 0.7% | 0.2% | 2.9% | 5 | 6.6% | 1.8% | 0.8% | 4.4% |
| **Urogenital** | 15 | 1.0% | 0 | . | . | . | . | 3 | 3.9% | 20.0% | 5.9% | 50.0% |
| **Resp OR fever** | 1,478 | 94.7% | 21 | 100% | 1.4% | 0.9% | 2.2% | 75 | 98.7% | 5.1% | 4.1% | 6.3% |
| **Respiratory** | 1,158 | 74.2% | 18 | 85.7% | 1.6% | 1.0% | 2.5% | 67 | 88.2% | 5.8% | 4.6% | 7.3% |
| **Fever** | 1,208 | 77.4% | 15 | 71.4% | 1.2% | 0.7% | 2.1% | 59 | 77.6% | 4.9% | 3.8% | 6.3% |
| **Diarrhoeal** | 459 | 29.4% | 5 | 23.8% | 1.1% | 0.5% | 2.6% | 17 | 22.4% | 3.7% | 2.3% | 5.9% |
| **Skin** | 75 | 4.8% | 0 | . | . | . | . | 1 | 1.3% | 1.3% | 0.2% | 9.1% |
| **Eye, ear, dental** | 52 | 3.3% | 1 | 4.8% | 1.9% | 0.3% | 13.0% | 0 | . | . | . | . |
| **Pain NOS** | 184 | 11.8% | 0 | . | . | . | . | 7 | 9.2% | 3.8% | 1.8% | 7.8% |
| **All other** | 328 | 21.0% | 11 | 52.4% | 3.4% | 1.9% | 6.0% | 25 | 32.9% | 7.6% | 5.2% | 11.1% |
| **Other unique** | 14 | 0.9% | 0 | . | . | . | . | 0 | . | . | . | . |
| ***Total <5 years*** | **1,561** | ***** | **21** | ***** | **1.3%** | **0.9%** | **2.1%** | **76** | ***** | **4.9%** | **3.9%** | **6.1%** |
| **5-14 years by presenting complaints** | | | | | | | | | | | | |
| **Abdominal** | 335 | 35.8% | 0 | . | . | . | . | 2 | 20.0% | 0.6% | 0.1% | 2.4% |
| **Urogenital** | 22 | 2.4% | 0 | . | . | . | . | 0 | . | . | . | . |
| **Resp OR fever** | 793 | 84.8% | 3 | 100% | 0.4% | 0.1% | 1.2% | 10 | 100% | 1.3% | 0.7% | 2.3% |
| **Respiratory** | 511 | 54.7% | 3 | 100% | 0.6% | 0.2% | 1.8% | 8 | 80.0% | 1.6% | 0.8% | 3.1% |
| **Fever** | 627 | 67.1% | 3 | 100% | 0.5% | 0.2% | 1.5% | 8 | 80.0% | 1.3% | 0.6% | 2.5% |
| **Diarrhoeal** | 172 | 18.4% | 0 | . | . | . | . | 2 | 20.0% | 1.2% | 0.3% | 4.6% |
| **Skin** | 32 | 3.4% | 0 | . | . | . | . | 0 | . | . | . | . |
| **Eye, ear, dental** | 35 | 3.7% | 0 | . | . | . | . | 0 | . | . | . | . |
| **Pain NOS** | 448 | 47.9% | 3 | 100% | 0.7% | 0.2% | 2.1% | 4 | 40.0% | 0.9% | 0.3% | 2.4% |
| **All other** | 175 | 18.7% | 1 | 33.3% | 0.6% | 0.1% | 4.0% | 4 | 40.0% | 2.3% | 0.9% | 6.0% |
| **Other unique** | 8 | 0.9% | 0 | . | . | . | . | 0 | . | . | . | . |
| ***Total 5-14 years*** | **935** | ***** | **3** | ***** | **0.3%** | **0.1%** | **1.0%** | **10** | ***** | **1.1%** | **0.6%** | **2.0%** |
| **15+ years by presenting complaints** | | | | | | | | | | | | |
| **Abdominal** | 1,249 | 38.0% | 0 | . | . | . | . | 4 | 23.5% | 0.3% | 0.1% | 0.9% |
| **Urogenital** | 311 | 9.5% | 0 | . | . | . | . | 1 | 5.9% | 0.3% | 0.0% | 2.3% |
| **Resp OR fever** | 2,015 | 61.4% | 2 | 66.7% | 0.1% | 0.0% | 0.4% | 11 | 64.7% | 0.5% | 0.3% | 1.0% |
| **Respiratory** | 1,200 | 36.5% | 2 | 66.7% | 0.2% | 0.0% | 0.7% | 7 | 41.2% | 0.6% | 0.3% | 1.2% |
| **Fever** | 1,330 | 40.5% | 1 | 33.3% | 0.1% | 0.0% | 0.5% | 6 | 35.3% | 0.5% | 0.2% | 1.0% |
| **Diarrhoeal** | 232 | 7.1% | 0 | . | . | . | . | 1 | 5.9% | 0.4% | 0.1% | 3.0% |
| **Skin** | 69 | 2.1% | 0 | . | . | . | . | 2 | 11.8% | 2.9% | 0.7% | 11.1% |
| **Eye, ear, dental** | 82 | 2.5% | 0 | . | . | . | . | 0 | . | . | . | . |
| **Pain NOS** | 2,165 | 65.9% | 2 | 66.7% | 0.1% | 0.0% | 0.4% | 11 | 64.7% | 0.5% | 0.3% | 0.9% |
| **All other** | 830 | 25.3% | 2 | 66.7% | 0.2% | 0.1% | 1.0% | 7 | 41.2% | 0.8% | 0.4% | 1.8% |
| **Other unique** | 63 | 1.9% | 0 | . | . | . | . | 1 | 5.9% | 1.6% | 0.2% | 10.8% |
| ***Total 15+ years*** | **3,284** | ***** | **3** | ***** | **0.1%** | **0.0%** | **0.3%** | **17** | ***** | **0.5%** | **0.3%** | **0.8%** |

| **HYPOXAEMIA PREVALENCE BY CLINICIAN DIAGNOSIS** | | | | | | | | | | | | |
| --- | --- | --- | --- | --- | --- | --- | --- | --- | --- | --- | --- | --- |
|  | **N** | **%** | **Severe hypoxaemia (SpO_2_<90%)** | | | | | **Moderate hypoxaemia (SpO_2_ 90-93%)** | | | | |
|  |  |  | **N** | **%** | **prevalence** | **95% CI** | | **N** | **%** | **prevalence** | **95% CI** | |
| **U5 years by diagnosis** | | | | | | | | | | | | |
| **ARI** | 811 | 52.0% | 13 | 61.9% | 1.6% | 0.9% | 2.7% | 53 | 69.7% | 6.5% | 5.0% | 8.5% |
| **ARI (no pneumonia)** | 678 | 43.4% | 6 | 28.6% | 0.9% | 0.4% | 2.0% | 35 | 46.1% | 5.2% | 3.7% | 7.1% |
| **“Pneumonia”** | 133 | 8.5% | 7 | 33.3% | 5.3% | 2.5% | 10.7% | 18 | 23.7% | 13.5% | 8.7% | 20.5% |
| **Diarrhoeal disease** | 243 | 15.6% | 3 | 14.3% | 1.2% | 0.4% | 3.8% | 10 | 13.2% | 4.1% | 2.2% | 7.5% |
| **Other GIT** | 8 | 0.5% | 0 | . | . | . | . | 0 | . | . | . | . |
| **Allergies** | 6 | 0.4% | 0 | . | . | . | . | 0 | . | . | . | . |
| **Ear infections** | 12 | 0.8% | 0 | . | . | . | . | 1 | 1.3% | 8.3% | 0.9% | 47.5% |
| **Injury** | 8 | 0.5% | 1 | 4.8% | 12.5% | 1.1% | 64.2% | 0 | . | . | . | . |
| **STI/Genital/UTI** | 7 | 0.4% | 0 | . | . | . | . | 1 | 1.3% | 14.3% | 1.2% | 70.1% |
| **Malaria** | 825 | 52.9% | 10 | 47.6% | 1.2% | 0.7% | 2.2% | 34 | 44.7% | 4.1% | 3.0% | 5.7% |
| **Sepsis** | 41 | 2.6% | 0 | . | . | . | . | 7 | 9.2% | 17.1% | 8.2% | 32.3% |
| **Helminths** | 41 | 2.6% | 0 | . | . | . | . | 1 | 1.3% | 2.4% | 0.3% | 16.2% |
| **Pregnancy** | 0 | . | . | . | . | . | . |  | . | . | . | . |
| **Skin** | 69 | 4.4% | 1 | 4.8% | 1.4% | 0.2% | 9.9% | 1 | 1.3% | 1.4% | 0.2% | 9.9% |
| **All other Dx** | 67 | 4.3% | 1 | 4.8% | 1.5% | 0.2% | 10.2% | 4 | 5.3% | 6.0% | 2.2% | 15.1% |
| **Other unique** | 23 | 1.5% | 0 | . | . | . | . | 0 | . | . | . | . |
| ***Total <5 years*** | **1,561** | ***** | **21** | ***** | **1.3%** | **0.9%** | **2.1%** | **76** | ***** | **4.9%** | **3.9%** | **6.1%** |
| **5-14 years by diagnosis** | | | | | | | | | | | | |
| **ARI** | 363 | 38.8% | 2 | 66.7% | 0.6% | 0.1% | 2.2% | 4 | 40.0% | 1.1% | 0.4% | 2.9% |
| **ARI (no pneumonia)** | 354 | 37.9% | 1 | 33.3% | 0.3% | 0.0% | 2.0% | 4 | 40.0% | 1.1% | 0.4% | 3.0% |
| **“Pneumonia”** | 9 | 1.0% | 1 | 33.3% | 11.1% | 1.1% | 59.1% | 0 | . | . | . | . |
| **Diarrhoeal disease** | 57 | 6.1% | 0 | . | . | . | . | 1 | 10.0% | 1.8% | 0.2% | 11.9% |
| **Other GIT** | 16 | 1.7% | 0 | . | . | . | . | 0 | . | . | . | . |
| **Allergies** | 7 | 0.7% | 0 | . | . | . | . | 0 | . | . | . | . |
| **Ear infections** | 10 | 1.1% | 0 | . | . | . | . | 0 | . | . | . | . |
| **Injury** | 6 | 0.6% | 0 | . | . | . | . | 0 | . | . |  | . |
| **STI/Genital/UTI** | 20 | 2.1% | 0 | . | . | . | . | 0 | . | . | . | . |
| **Malaria** | 538 | 57.5% | 1 | 33.3% | 0.2% | 0.0% | 1.3% | 5 | 50.0% | 0.9% | 0.4% | 2.2% |
| **Sepsis** | 14 | 1.5% | 0 | . | . | . | . | 0 | . | . | . | . |
| **Helminths** | 46 | 4.9% | 0 | . | . | . | . | 0 | . | . | . | . |
| **Pregnancy** | 0 | . | . | . | . | . | . | . | . | . | . | . |
| **Skin** | 27 | 2.9% | 0 | . | . | . | . | 0 | . | . | . | . |
| **All other Dx** | 59 | 6.3% | 2 | 66.7% | 3.4% | 0.8% | 12.9% | 2 | 20.0% | 3.4% | 0.8% | 12.9% |
| **Other unique** | 37 | 4.0% | 1 | 33.3% | 2.7% | 0.4% | 17.8% | 1 | 10.0% | 2.7% | 0.4% | 17.8% |
| ***Total 5-14 years*** | **935** | ***** | **3** | ***** | **0.3%** | **0.1%** | **1.0%** | **10** |  | **1.1%** | **0.6%** | **2.0%** |
| **15+ years by diagnosis** | | | | | | | | | | | | |
| **ARI** | 1,004 | 30.6% | 0 | . | . | . | . | 6 | 35.3% | 0.6% | 0.3% | 1.3% |
| **ARI (no pneumonia)** | 990 | 30.1% | 0 | . | . | . | . | 6 | 35.3% | 0.6% | 0.3% | 1.3% |
| **“Pneumonia”** | 14 | 0.4% | 0 | . | . | . | . | 0 | . | . | . | . |
| **Diarrhoeal disease** | 113 | 3.4% | 0 | . | . | . | . | 0 | . | . | . | . |
| **Other GIT** | 375 | 11.4% | 0 | . | . | . | . | 2 | 11.8% | 0.5% | 0.1% | 2.1% |
| **Allergies** | 30 | 0.9% | 0 | . | . | . | . | 1 | 5.9% | 3.3% | 0.4% | 21.6% |
| **Ear infections** | 23 | 0.7% | 0 | . | . | . | . | 0 | . | . | . | . |
| **Injury** | 48 | 1.5% | 0 | . | . | . | . | 0 | . | . | . | . |
| **STI/Genital/UTI** | 620 | 18.9% | 0 | . | . | . | . | 3 | 17.6% | 0.5% | 0.2% | 1.5% |
| **Malaria** | 965 | 29.4% | 1 | 33.3% | 0.1% | 0.0% | 0.7% | 3 | 17.6% | 0.3% | 0.1% | 1.0% |
| **Sepsis** | 56 | 1.7% | 0 | . | . | . | . | 0 | . | . | . | . |
| **Helminths** | 66 | 2.0% | 0 | . | . | . | . | 1 | 5.9% | 1.5% | 0.2% | 10.3% |
| **Pregnancy** | 41 | 1.2% | 0 | . | . | . | . | 0 | . | . | . | . |
| **Skin** | 63 | 1.9% | 0 | . | . | . | . | 1 | 5.9% | 1.6% | 0.2% | 10.8% |
| **All other Dx** | 588 | 17.9% | 1 | 33.3% | 0.2% | 0.0% | 1.2% | 4 | 23.5% | 0.7% | 0.3% | 1.8% |
| **Other unique** | 373 | 11.4% | 1 | 33.3% | 0.3% | 0.0% | 1.9% | 2 | 11.8% | 0.5% | 0.1% | 2.1% |
| ***Total 15+ years*** | **3,284** | ***** | **3** |  | **0.1%** | **0.0%** | **0.3%** | **17** | ***** | **0.5%** | **0.3%** | **0.8%** |

ARI = acute respiratory infection; CI = confidence interval; Dx = diagnoses; GIT = gastrointestinal tract; STI = sexually transmitted infection; UTI = urinary tract infection;
