# Supplementary material for: Hypoxaemia prevalence and management among children and adults presenting to primary care facilities in Uganda: A prospective cohort study
Source: PLOS Glob Public Health. 2022 Apr 22;2(4):e0000352. doi: 10.1371/journal.pgph.0000352 (PMC10022140; doi:10.1371/journal.pgph.0000352)
Supplement: S3 Table — Final model = after backward stepwise selection using P<0.05 as the primary determinant for exclusion at each step; CI = confidence interval; ICC–inter-cluster correlation coefficient. (DOCX) [file pgph.0000352.s005.docx]

## S3 TABLE: PREDICTORS OF HYPOXAEMIA

**Predictors of hypoxaemia among children, adolescents, and adults presenting to HCIII facilities in Uganda, using mixed-effects logistic regression**

| ***Final model*** | **Under 5** | | | | **Under 15** | | | | **5-14 years** | | | | **15+** | | | | | |
| --- | --- | --- | --- | --- | --- | --- | --- | --- | --- | --- | --- | --- | --- | --- | --- | --- | --- | --- |
|  | **aOR** | **95%** | **CI** | **p** | **aOR** | **95%** | **CI** | **p** | **aOR** | **95%** | **CI** | **p** | **aOR** | **95%** | **CI** | **p** |  |  |
| **Age** | 0.66 | 0.54 | 0.79 | 0.00 | 0.79 | 0.73 | 0.86 | 0.00 | . | . |  | . | 1.05 | 1.02 | 1.07 | 0.00 |  |  |
| **Respiratory complaints** | 2.46 | 1.30 | 4.66 | 0.01 | 2.85 | 1.59 | 5.12 | 0.00 | 5.11 | 1.10 | 23.73 | 0.04 | . | . |  | . |  |  |
|  | **ICC** | **95%** | **CI** | | **ICC** | **95%** | **CI** | | **ICC** | **95%** | **CI** | | **ICC** | **95%** | **CI** | | |  |
| **Facility** | 0.21 | 0.09 | 0.42 |  | 0.18 | 0.08 | 0.36 |  | 0.18 | 0.02 | 0.69 |  | 0.31 | 0.09 | 0.67 |  |  |  |
|  |  |  |  |  |  |  |  |  |  |  |  |  |  |  |  |  |  |  |
| ***Full model*** | **Under 5 years** | | | | **Under 15 years** | | | | **5-14 years** | | | | **15+ years** | | | | | |
|  | **aOR** | **95%** | **CI** | **p** | **aOR** | **95%** | **CI** | **p** | **aOR** | **95%** | **CI** | **p** | **aOR** | **95%** | **CI** | **p** |  |  |
| **Region** | 0.43 | 0.17 | 1.09 | 0.08 | 0.60 | 0.26 | 1.37 | 0.22 | 1.82 | 0.41 | 8.00 | 0.43 | 0.58 | 0.13 | 2.60 | 0.47 |  |  |
| **Age (years)** | 0.67 | 0.55 | 0.81 | 0.00 | 0.80 | 0.73 | 0.88 | 0.00 | 1.10 | 0.89 | 1.37 | 0.38 | 1.05 | 1.02 | 1.07 | 0.00 |  |  |
| **Sex** | 1.22 | 0.79 | 1.88 | 0.36 | 1.05 | 0.70 | 1.56 | 0.82 | 0.42 | 0.13 | 1.35 | 0.15 | 1.27 | 0.43 | 3.80 | 0.66 |  |  |
| **Abdominal complaints** | 0.61 | 0.27 | 1.39 | 0.24 | 0.52 | 0.25 | 1.07 | 0.08 | 0.51 | 0.11 | 2.49 | 0.41 | 0.46 | 0.14 | 1.49 | 0.20 |  |  |
| **Urogenital complaints** | . | . |  | . | . | . |  | . | . | . |  | . | 0.65 | 0.08 | 5.32 | 0.69 |  |  |
| **Respiratory complaints** | 2.24 | 1.16 | 4.30 | 0.02 | 2.53 | 1.39 | 4.61 | 0.00 | 5.43 | 1.10 | 26.85 | 0.04 | 1.13 | 0.44 | 2.92 | 0.80 |  |  |
| **Diarrhoea and vomiting** | 0.75 | 0.44 | 1.25 | 0.27 | 0.79 | 0.49 | 1.29 | 0.35 | 1.36 | 0.27 | 6.96 | 0.71 | 0.90 | 0.11 | 7.18 | 0.92 |  |  |
| **Fever and chills** | 0.87 | 0.51 | 1.48 | 0.60 | 1.01 | 0.61 | 1.66 | 0.97 | 3.20 | 0.66 | 15.59 | 0.15 | 0.74 | 0.28 | 1.98 | 0.55 |  |  |
| **Pain** | 0.83 | 0.36 | 1.93 | 0.67 | 0.95 | 0.50 | 1.79 | 0.86 | 1.84 | 0.56 | 5.99 | 0.31 | 0.60 | 0.21 | 1.69 | 0.33 |  |  |
| **Intercept** | 0.67 | 0.02 | 18.15 | 0.81 | 0.19 | 0.01 | 3.54 | 0.26 | 0.00 | 0.00 | 0.11 | 0.01 | 0.00 | 0.00 | 1.44 | 0.07 |  |  |
|  | **ICC** | **95%** | **CI** | | **ICC** | **95%** | **CI** | | **ICC** | **95%** | **CI** | | **ICC** | **95%** | **CI** | | |  |
| **Facility** | 0.18 | 0.07 | 0.39 |  | 0.15 | 0.06 | 0.34 | | 0.22 | 0.22 | 0.22 |  | 0.29 | 0.08 | 0.66 | | |  |

Final model = after backward stepwise selection using P<0.05 as the primary determinant for exclusion at each step; CI = confidence interval; ICC – inter-cluster correlation coefficient;
