# Supplementary material for: Hypoxaemia prevalence and management among children and adults presenting to primary care facilities in Uganda: A prospective cohort study
Source: PLOS Glob Public Health. 2022 Apr 22;2(4):e0000352. doi: 10.1371/journal.pgph.0000352 (PMC10022140; doi:10.1371/journal.pgph.0000352)
Supplement: S4 Table — Final model = after backward stepwise selection using P<0.05 as the primary determinant for exclusion at each step; CI = confidence interval; ICC–inter-cluster correlation coefficient. (DOCX) [file pgph.0000352.s006.docx]

## S4 TABLE: Predictors of referral among children, adolescents, and adults presenting to HCIII facilities in Uganda, using mixed-effects logistic regression

| ***Final model*** | **Under 5 years** | | | | **Under 15 years** | | | | **5-14 years** | | | | **15+ years** | | | | | |
| --- | --- | --- | --- | --- | --- | --- | --- | --- | --- | --- | --- | --- | --- | --- | --- | --- | --- | --- |
|  | **aOR** | **95%** | **CI** | **p** | **aOR** | **95%** | **CI** | **p** | **aOR** | **95%** | **CI** | **p** | **aOR** | **95%** | **CI** | **p** |  |  |
| **Region** | 18.5 | 1.03 | 331.8 | 0.05 |  |  |  |  |  |  |  |  | 19.5 | 3.69 | 102.5 | 0.00 |  |  |
| **Age (years)** | 0.24 | 0.07 | 0.88 | 0.03 |  |  |  |  |  |  |  |  | 1.03 | 1.00 | 1.05 | 0.03 |  |  |
| **Sex** |  |  |  |  |  |  |  |  |  |  |  |  |  |  |  |  |  |  |
| **SpO_2_<94%** | 960 | 30.7 | 29973 | 0.00 | 41.0 | 13.9 | 121.2 | 0.00 | 19.1 | 1.98 | 183.6 | 0.01 | 58.0 | 9.95 | 338.6 | 0.00 |  |  |
| **Abdominal complaints** | 16.0 | 1.22 | 211.1 | 0.04 |  |  |  |  |  |  |  |  |  |  |  |  |  |  |
| **Urogenital complaints** | . | . |  | . |  |  |  |  |  |  |  |  |  |  |  |  |  |  |
| **Respiratory complaints** | 0.06 | 0.01 | 0.61 | 0.02 |  |  |  |  |  |  |  |  |  |  |  |  |  |  |
| **Diarrhoea and vomiting** |  |  |  |  |  |  |  |  |  |  |  |  |  |  |  |  |  |  |
| **Skin complaints** |  |  |  |  |  |  |  |  |  |  |  |  |  |  |  |  |  |  |
| **Fever and chills** |  |  |  |  |  |  |  |  |  |  |  |  |  |  |  |  |  |  |
| **Pain NOS** |  |  |  |  |  |  |  |  |  |  |  |  |  |  |  |  |  |  |
|  | **ICC** | **95%** | **CI** | | **ICC** | **95%** | **CI** | | **ICC** | **95%** | **CI** | | **ICC** | **95%** | **CI** | | |  |
| **Facility** | 0.47 | 0.13 | 0.84 |  | 0.17 | 0.03 | 0.57 | | NA |  |  |  | 0.09 | 0.01 | 0.54 | | |  |

Final model = after backward stepwise selection using P<0.05 as the primary determinant for exclusion at each step; CI = confidence interval; ICC – inter-cluster correlation coefficient;
