# Supplementary material for: Hypoxaemia prevalence and management among children and adults presenting to primary care facilities in Uganda: A prospective cohort study
Source: PLOS Glob Public Health. 2022 Apr 22;2(4):e0000352. doi: 10.1371/journal.pgph.0000352 (PMC10022140; doi:10.1371/journal.pgph.0000352)
Supplement: Attachment — Submitted filename: Response to Reviewers.docx [file pgph.0000352.s007.docx]

# Response to Reviewers/Editor

**PLOS Global Public Health: PGPH-D-21-01118** - Hypoxaemia prevalence and management among children and adults presenting to primary care facilities in Uganda: a prospective cohort study.

We thank the Reviewers and Editors for their comments and time. Your suggestions have helped add clarity and strength and we hope you are pleased with the revisions detailed below.

Journal Requirements:

1. Please include a complete copy of PLOS’ questionnaire on inclusivity in global research in your revised manuscript. Our policy for research in this area aims to improve transparency in the reporting of research performed outside of researchers’ own country or community. The policy applies to researchers who have travelled to a different country to conduct research, research with Indigenous populations or their lands, and research on cultural artefacts. The questionnaire can also be requested at the journal’s discretion for any other submissions, even if these conditions are not met.  Please find more information on the policy and a link to download a blank copy of the questionnaire here: <https://journals.plos.org/plosone/s/best-practices-in-research-reporting>. Please upload a completed version of your questionnaire as Supporting Information when you resubmit your manuscript.

Completed and uploaded as a supplemental file as requested. This research was led by Ugandan implementers with senior oversight by Ugandan academics. Seven of the 11 named authors are Ugandan residents based full-time in Uganda, including the senior authors. International staff provided program and/or academic oversight, including responsibility for protocol development, data analysis and reporting.

2. Please provide separate figure files in .tif or .eps format only, and remove any figures embedded in your manuscript file.

For more information about figure files please see our guidelines: 
<https://journals.plos.org/globalpublichealth/s/figures>

Figures provided as separate image files in .tif format in accordance with guidelines.

3. We noticed that you used "unpublished data" in the manuscript. We do not allow these references, as the PLOS data access policy requires that all data be either published with the manuscript or made available in a publicly accessible database. Please either remove these references, or amend the supplementary material to include the referenced data.

Thank you. We have updated the referencing to indicate the source reports and provided all data in the included Table.

4. Please update the completed 'Competing Interests' statement, including any COIs declared by your co-authors. If you have no competing interests to declare, please state "The authors have declared that no competing interests exist". Otherwise please declare all competing interests beginning with the statement "I have read the journal's policy and the authors of this manuscript have the following competing interests:"

Thank you. We have updated the COI section accordingly.

5. We have noticed that you have uploaded supporting information but you have not included a list of legends.  Please add a full list of legends for all supporting information files (including figures, table and data files) after the references list.

Thank you. All supplemental material is contained within the single Supplemental Material document. We have updated the figure and table legends within this document and included a Table of Contents for easy navigation. We have updated the reference text in the manuscript to specify which supplemental table/figure.

6. Please amend your detailed Financial Disclosure statement. This is published with the article, therefore should be completed in full sentences and contain the exact wording you wish to be published.
 
ii). State the initials, alongside each funding source, of each author to receive each grant.
iii). State what role the funders took in the study. If the funders had no role in your study, please state: “The funders had no role in study design, data collection and analysis, decision to publish, or preparation of the manuscript.”

Thank you. We have updated the financial disclosure statement accordingly. The funding was awarded to an institution (not individual grantees).

5. Review Comments to the Author

Reviewer #1: Abstract:
• Intro and Para 1 of main text Introduction – It is stated that: ‘Low blood oxygen levels (hypoxaemia) are common among children and adults admitted to hospital 30 and it increases their risk of death 5-fold and thus requires prompt detection and treatment.’ – I think the data indicate that hypoxaemia is associated with a 5 fold increase in the odds of death; stating causality in such strong terms would suggest the risk of death can be reduced 5 fold by correcting hypoxaemia which is not as far as I am aware proven.
Thank you. We have rephrased to be more precise.

*Low blood oxygen levels (hypoxaemia) are common among children and adults admitted to hospital and associated with a 5-fold increase in the odds of death and thus requires prompt detection and treatment.*

• Secondary outcome in the abstract is stated as “severe/moderate hypoxaemia (SpO2 90-93%) by 13 age/sex/complaint” – this wording is confusing, is this all Sp02<94%?
Thank you. We have rephrased to clarify.

*Secondary outcomes: severe (SpO_2_<90%) and moderate hypoxaemia (SpO_2_ 90-93%) prevalence by age/sex/complaint; number of children with hypoxaemia referred, admitted and recovered.*

• Conclusion – the recommendations include “Effectiveness, acceptability, and feasibility of pulse oximetry 26 and oxygen therapy for primary care should be investigated in implementation trials.” – Do the authors think that costs and consequences to families and health systems should also be assessed as there may well be implications from referrals that do not improve clinical outcomes?
Yes, absolutely. Economic analysis of pulse oximetry is important from both provider and user/patient perspectives. We have added a comment to that effect, and flagged the cost/economic aspects of pulse oximetry implementation more clearly in Discussion.

General comments:
• No mention is made of whether the sites of this study are equipped to manage asthma in any way if they feel this diagnosis is present?
HCIII facilities are expected to manage mild to moderate asthma including mildly and moderately persistent asthma in children and adults with inhaled bronchodilators and referral to level IV health center or hospital where necessary. We did not directly assess what asthma services were being provided at participating facilities. We did not collect independent clinical data to verify HCW diagnoses of pneumonia, asthma, COPD (or other conditions) and have added a note to this in Limitations.

*We relied on HCW diagnostic classifications and did not collect independent data to validate diagnoses or distinguish between different respiratory illnesses (e.g. pneumonia, bronchiolitis, asthma, chronic obstructive pulmonary disease).*

• Throughout the paper it is sometimes hard to keep track of what is meant by hypoxaemia, sometimes the term is used alone, sometimes qualified by either severe or moderate. In general it might be better to use the pulse oximetry values throughout rather then the term hypoxaemia even if qualified by an adjective. In a similar vein it would be helpful to carefully use <94% to mean all those with a saturation lower than this or 90-93% for the specific group with those values (see Fig3 title for example).
Thank you. We have revised for clarity, labelling the SpO_2_ range in every instance and using the lone term ‘hypoxaemia’ only in the generic sense. We have clearly defined the severe <90% and moderate 90-93% ranges and rationale in Methods, and reviewed to ensure consistency throughout.

• In the discussion section where results are repeated as % values please continue to indicate the 95% CI around the estimates.
Accepted suggestion. Added 95% confidence intervals.

• The section in the discussion on hypoxaemia in children repeats much of what has been stated in the results and what is provided in Table 3 is stated in the narrative text too. This section of the discussion could be made more concise and clearly state what added points are being made.
Accepted suggestion. We have limited repetition to a single sentence of major results, leading into comparison with existing literature and comments on interpretation. We have reduced repetition between Table 3 and the text.

Specific comments
o Line 303 – “We found that moderate or severe hypoxaemia was relatively common among children U5 presenting to primary care facilities (1.3% had SpO2<90% and 4.9% had SpO2 90-93%) but uncommon among older children aged 5-14 years (0.3% SpO2<90%, 1.4% SpO2 90-93%). Assuming similar prevalence nationally and throughout the year, this equates to >40,000 children U5 presenting to HCIIIs in Uganda with hypoxaemia annually (including ~8,700 with severe hypoxaemia) – most of whom are not currently identified, treated, or referred.” - While the findings restated here are those found in the text the implicit messaging here is that those with oxygen saturation of 90 to 93% should be ‘identified, treated and referred’. Is this the author’s intention and if so what evidence supports this course of action? This might result in a 300% increase in respiratory referrals? Given recent findings on the potential for error in dark skins this challenge seems worth a mention here.
Thank you. We did not intend to draw any specific reference here, and the identified/treated/referred statement was intended to apply primarily to the severe hypoxaemia SpO2<90% group (linking directly to our finding that the majority of children with severe hypoxaemia were not referred and did not go to hospital). We have revised for clarity.

*Assuming similar prevalence nationally and throughout the year, this equates to >40,000 children U5 presenting to HCIIIs in Uganda with SpO_2_<94% annually, including ~8,700 with severe hypoxaemia (SpO_2_<90%) – of whom <50% are currently identified, treated, or referred.*

• Line 339 – the 1st reference is given to support this statement ‘This risk of death is proportional to the degree of hypoxaemia with higher risk of death even among children with moderate hypoxaemia (SpO2 90-93%)’ but the original reference does not provide a clear articulation of this apparent risk and is from an inpatient population (including neonates) with much higher rates of hypoxaemia overall and much higher risks of death. Please clarify / correct the statement or remove it.
Thank you. See Fig 2 in the cited paper (the graph disaggregates data by age showing much higher predicted probability of death with low SpO_2_ across all age strata). Previous studies have shown similar in particular sub-populations but this is the largest and most comprehensive example. Regarding the specific cohort with SpO2 90-93%, this is also reflected in other studies on particular sub-populations (including primary care child pneumonia) and these references have now been added.

• Line 345 – “Our study was not powered to detect differences in mortality, but it is concerning that a significant proportion of children who were not referred were subsequently taken to a referral facility by caregivers, required admission, and had not fully recovered by day 7” – it is probably helpful not to use the term ‘significant’ here – please state the actual numbers meant and perhaps caveat this statement with the fact that none of these children as far as I can tell died while it seems some of those referred or admitted had also not recovered? It is also worth remembering that there are real costs borne by families from referral that are both direct and indirect (eg loss of income). In essence pulse oximetry in primary care is being proposed as a screening tool and therefore we have to carefully consider the consequences and costs of both false positives and false negatives especially in situations where the true prevalence can be very low. Given that the readings taken in this study were done by supernumerary, trained staff who took up to 3 readings it is hard to know what these false positive and false negative % would be in ‘real life’ and what effects of routine oximetry would have on working practices. (These issues are relevant to later discussions on generalisability)
Thank you. We agree this is unclear as currently stated and without substantial elaboration so we have deleted this statement entirely.

• Line 388 – this statement is made – “Second, adoption and sustained use of pulse oximetry relies on HCWs being convinced that it provides a benefit”. – Would the authors agree that to recommend adoption and sustained use of pulse oximetry we first need to provide good evidence of its benefit before asking health workers to take it on?
Yes, and one of our major conclusions is the need for intentional studies looking at effectiveness, appropriateness, feasibility, and costs of implementing pulse oximetry in primary care settings. This study does not provide evidence on effectiveness at all – rather it provides evidence to support early implementation studies. I think we share a real concern that norm-setting agencies (e.g. WHO) are rushing to implement something for which the implications, and how to most effectively do it, are poorly understood. The next step in our program of work is to use what we have learnt here to design and test a strategy for using pulse oximetry in primary care in Uganda, using an implementation research lens with a component of cost-effectiveness analysis from a societal perspective.

• Line 395 – “Third, the hypoxaemia threshold for clinical concern and referral should probably be more inclusive than the threshold for providing oxygen therapy.” – Would the authors like to comment on the variations in guidance and practice in high income countries with regard to when oxygen saturation values indicate clinical concern and how this might vary with possible disease aetiology and age (eg bronchiolitis / RSV). It is also unclear to me what is actual meant by SpO2 from 90-93% being ‘an indication for strong clinical concern and consideration for referral and admission’. Are the authors proposing this threshold replaces current guidance or not at primary care level where health workers with limited skills operate?
Thank you. These are not intended to be read as “recommendations”, but as “areas that warrant consideration and further exploration”. So no, we are not advocating for any change in guidelines. We are simply saying that, aside from identifying which patients need oxygen, pulse oximetry is a useful tool in identifying those at greater risk of death – and this may be the most important role in primary care: identifying severely unwell patients we should be worried about. We know that mortality is predicted by decreasing SpO2 below 94% (see earlier comments) and this study shows that there are a large number of children in that ‘at-risk’ group. We know that many of these children will not be picked up by other clinical signs (e.g. WHO general danger signs). That doesn’t necessarily mean all these children need urgent referral and admission but it does offer the opportunity to recognise them as at-risk, and provide appropriate safeguards. In all likelihood the most appropriate cut-offs and actions will be context-specific and that is why we need more operational research from different contexts to help local ministries of health to make reasonable decisions. We have added a statement to this effect, with references:

“Previous studies have found that cut-points of <92% or <94% provide better discrimination for severe disease (and poor outcome) than <90%, but such decisions for guidelines must also consider the local health system and individual clinical context and economic implications from a societal perspective.”

• Line 403 – Pulse oximetry may appear to be a simple practice but what are the challenges in providing and sustaining its use across nearly 1000 HCIII in Uganda – how has progress been in providing this technology in HCIV or district general hospitals in Uganda?
Good question. This research is part of a broader MOH-CHAI program to improve pulse oximetry and oxygen use at HCIV and district general hospitals in catchment areas of Jinja and Mubende Regional Referral Hospitals (the target areas of this study). This program of work was intended to develop lessons from these regions to push forward the National Scale-up of Medical Oxygen Implementation Plan nationally – and the COVID19 pandemic response has accelerated this, with national equipment distribution and training occurring during 2020-21. Interim program data from the study areas show dramatic improvements in pulse oximetry and oxygen availability and patient coverage at HCIV and hospitals over this time and we are learning many practical lessons through ongoing evaluation. Early implementation of pulse oximetry in selected HCIIIs is beginning now, informed by findings from the current study, and evaluation will answer more of the questions about feasibility at this level of care. That said, the authors would like to note that inadequate investment in diagnostic capacity of healthcare facilities (level III and above) for illnesses intended to be managed at that level of care in Uganda has been a perennial problem. It is therefore imperative for such studies to investigate implementation questions and provide appropriate evidence for engagement with decision makers for more resource allocation and prioritization.

Minor
• Line 198 – is ‘respiratory’ a presenting complaint? Seems words missing?
Yes, these are all categories of complaints.
• Line 227 - Hypoxaemia prevalence was most common among children diagnosed with ARI (8.1%, 6.4 to 10.2), malaria (5.3%, 4.0 to 7.1), or sepsis (17.1%, 8.2 to 32.3), and particularly high for those diagnosed with pneumonia (18.8%, 13.0 to 26.4) – this is a little hard to interpret since the diagnosis was made with knowledge of the pulse oximetry value?
Noted. Pulse oximetry results may have been used by HCWs to formulate diagnosis, and this would be appropriate and consistent with IMCI guidelines. We did not seek to evaluate whether diagnosis changed because of pulse oximetry (but this will be part of the early implementation evaluation).

• I am not sure this ‘result’ has much meaning - Oxygen was rarely available and was provided to only 3 patients (0.06%). A severely hypoxaemic (SpO2 61%) infant with febrile respiratory illness was the only patient with SpO2<90% who received oxygen therapy (1/27, 3.7%). Two other patients with SpO2 93% and 98% were recorded as receiving oxygen – is this because only one place had oxygen for only some of the time or all places had oxygen rarely but didn’t use it when they could?
Revised for clarity: Oxygen was available in very few facilities and used for only 3 patients (0.06%) during the study period.

• Line 269 – were the 6 admitted overnight the same as those who were referred to a larger facility or who went elsewhere for care?
No, half those admitted were from direct referrals and half from those who had not initially been referred. But small numbers and difficult to interpret such details and we did not capture data on why they were or were not admitted.

• Line 298 - Current referral practices are missing a significant number of severely unwell children with hypoxaemia and pulse oximetry may be a useful tool in helping HCWs prioritise patients and facilitate referral. – However, the majority of those with severe hypoxaemia but not referred recovered without referral care?
Thank you for this observation. Indeed, the authors agree that the majority of those with severe hypoxaemia but not referred recovered without referral care – and this fits with our understanding of hypoxaemia and acute respiratory infection (i.e. hypoxaemia increases risk of death but it is not universally fatal and many young children have viral respiratory infections which will resolve without antibiotics). It is also important to note that the study was not designed (or powered) to detect differences in clinical outcomes.

• Line 320 - However, the relatively low pneumonia diagnosis case numbers suggest significant under-diagnosis. – What is the justification for this statement?
Thank you. Revised: “may suggest under-diagnosis of WHO-defined pneumonia”. HCWs assigned a diagnosis of ‘pneumonia’ to 8% of children U5, while 52% were diagnosed “ARI” and 53% “malaria”. Broadly speaking I’d expect at least 10-15% of acutely unwell children U5 to meet WHO pneumonia criteria in primary care settings in SSA. We know that pneumonia-malaria diagnosis is difficult, with general tendency to under-diagnose pneumonia in children with positive malaria tests in malaria-endemic settings. More specifically: upper ARI should not cause hypoxaemia – and yet there were equal numbers of children with hypoxaemia diagnosed “ARI” as “ARI-pneumonia”.

• Line 33 & 332 – presumably variability in the aetiology of respiratory infection and/or asthma also influences apparent rates of hypoxaemia?
Good point. Revised to include aetiology.

Reviewer #2: This study is unique because it explores hypoxaemia in primary care and includes both adults and children. Most studies in low income countries to date have studied hypoxaemia in inpatients - at secondary and tertiary referral - with a focus either on children or adults. I feel that this study will be a useful addition to the existing literature and will make an important contribution toward scaling up pulse oximetry use and promoting rational oxygen therapy in low resource settings.
Overall this was a clear and well-presented paper, reporting on the prevalence of hypoxaemia in primary care facilities in Uganda. I have the following comments which may require the authors’ response.
Abstract
1. The abstract is well written and contains pertinent information about the study.
I would suggest that the authors consider reporting the numbers of participants in the study in the abstract because at present a reader will have no idea about the size of this study by reading through the abstract alone.
Accepted suggestion: “We included 1561 children U5, 935 children 5-14 years, and 3284 adolescents/adults 15+ years.”

2. The methods in the abstract mentions that blood oxygen level was assessed but does not indicate how this assessment was done – it will be helpful to mention pulse oximetry in the abstract methods because the conclusion of the study is on pulse oximetry yet there is no mention of pulse oximetry before this conclusion.
Accepted suggestion: “Clinical data collectors used handheld pulse oximeters to measure blood oxygen level (SpO_2_)…”

3. Information on follow up is pertinent in a cohort study and it is important for the authors to provide some detail (at least for the abstract an indication of period of follow up would be useful).
Accepted suggestion: “We followed up children aged under 15 years with SpO_2_<93% by phone after 7 days to determine if the patient had attended another health facility, been admitted, or recovered.”

Introduction
4. The introduction provides a clear scientific background and rationale for the study, and the objectives are stated.
Thank you for this observation.

5. The authors state that they are aware of only three studies that report hypoxemia prevalence in primary care settings. However, they proceed to provide references for two studies (Reference 11 from Ethiopia and 14 from Malawi). Did they forget to reference the study from The Gambia?
6. I also conducted a search and found the study by Blanc et al on pulse oximetry in young children with severe illness in outpatient clinics in Papua New Guinea (<https://doi.org/10.1371/journal.pone.0213937>). It would be good to get the authors’ perspective on why this study and possibly others were not considered as relevant – this may require the authors to qualify their statement as to why they feel only the three studies they identified address the issue at hand. For example, in the discussion you suggest that there are no published studies on hypoxemia prevalence among general populations presenting to outpatient. I suggest that such statement in the introduction and discussion are made consistent and while avoiding broad, unqualified statements.
The authors are grateful for this observation.. This was an unfortunate oversight. The Blanc paper did come up in our (ongoing) systematic review but we had failed to reference it here. We have added this and revised the Intro and Discussion to ensure consistency and avoid broad, unqualified statements.

Methods
7. The study design in presented as a prospective cohort study. My appraisal of the study design is that the prospective cohort design applies to only 87 children with severe and moderate hypoxaemia (line 264 – 291), out of a total of 5813 participants in the study. Most participants contribute only cross-sectional data used to determine prevalence and predictors of hypoxaemia. This detail is obscured in the title and methods and I feel that this may have an important bearing on the classification of the study design. The authors may want to give a clearer description of the study design highlighting these issues.
Your interpretation is correct. All acutely unwell children, adolescents, and adults were included in the first cross-sectional data collection and only children <15 years with SpO2<93% were followed up for subsequent data collection (the prospective cohort). We have revised the methods section, including:

“We conducted a cross-sectional assessment of hypoxaemia among children, adolescents and adults, and prospectively followed a cohort of children with hypoxaemia to assess hypoxaemia management and outcomes.”

We have revised the abstract, including key results from the cohort:

“We followed 87 children aged under 15 years with SpO_2_<93%, with complete data for 61 (70%), finding low rates of referral (6/61, 10%), hospital attendance (10/61, 16%), and admission (6/61, 10%) with most (44/61, 72%) fully recovered at day 7. Barriers to referral included caregiver belief it was unnecessary (42/51, 82%), cost (8/51, 16%), and distance or lack of transport (3/51, 6%).”

8. For a prospective cohort study I feel that the authors did not provide pertinent details that are required for a cohort design. The information on the follow up period is very limited, and is mentioned only in one sentence in data collection with respect to consenting for data collection (line 139 i.e. … patients/caregivers were also invited to consent to a follow up phone call after 7 days…). In prospective cohorts follow up is planned as part of the design and should be well described within the study design and or participants’ sections.
Thank you. We have revised to ensure reporting is in line with the STROBE reporting guidelines and clarify the cohort that was included in follow up.

9. The authors provide a detailed description of the study setting including levels of health facilities in Uganda and typical health services provided. A potentially relevant aspect of study settings in hypoxaemia studies is altitude because of its association with oxygen levels in blood. The authors could consider providing details of the altitude of study sites.
Selection of health facilities is described in detail.
Thank you. We have added detail on altitude – both regions are low-altitude ~1100-1400m elevation above sea level.

10. The sample size calculation assumed that 3500 children under 5 would be available in the study but the study ended up recruiting about half of this number. What is the implication of this sample size that was obtained after recruitment?
Thank you. You are correct. Sample size achieved was lower than anticipated and prevalence higher than predicted. As evident from the confidence intervals, this resulted in slightly less precision in prevalence estimates.

11. You use two definitions of severe hypoxemia in different sections of the manuscript i.e. SpO2 < 93 and SpO2 < 94. Please check these definitions that appear to be inconsistent.
Thank you. Please see response to Reviewer 1. Revised for clarity throughout.

12. The multilevel random effect model is appropriate for the type of data in this analysis. The description of the model building is not adequate because the author simply states they used backward stepwise selection – additional details on the variable selection (how you determined the least significant variables at each step) and the stopping rule will be useful.
Thank you. We have added detail describing the primary use of individual variable P-values to determine which variable would be dropped at each step (as we were primarily interested in the predictive value of individual variables rather than the overall model fit).

Results
13. A significant number of exclusion is in children who were unaccompanied during the visit to facilities. It will be helpful to comment on this group as it is a possible source of selection bias.
Some of the data that you present in text in the results section do not appear in the tables, for example on the prevalence of hypoxemia you report that “Overall hypoxaemia (SpO2<94%) prevalence among children and adults presenting with acute illness to HCIII primary care facilities in Uganda was 2.2% (95% CI 1.9 to 2.7) (Table 2).” However, these overall results do not appear in table 2. This is the case for several other results sections where you report overall results in text. Please consider adding a column for the overall results in Table2.
We removed the ‘total’ column from the main results tables based on previous internal reviewer feedback as it made the tables too cumbersome. Aside from the confidence intervals, all the estimates can be derived directly from the data in Table 2 (the numbers and % simply sum).

14. Considering the percentages in Table 2, I am not completely in agreement that hypoxemia showed a bimodal distribution with age.
Please also see figure in Supplemental material. See below. Older adults are under-represented so the numbers with hypoxaemia hide the relatively high rates of hypoxaemia.

15. The authors should consider briefly describing the WHO IMCI guidelines for performing pulse oximetry so as to provide context for readers who are not familiar with the guidelines to interpret the findings in this study.
Thank you for this observation. We have taken it into account and revised the relevant section of the manuscript in the introduction to make it more clear:

“Current WHO primary care guidelines recommend pulse oximetry “if available” for children U5 with respiratory complaints, with recommendation for referral if SpO_2_<90%.(9) However, pulse oximetry availability and use in primary care settings is rare.(10-12)”

16. The analysis of pathway of care was presented quite effectively using Figure 3.
Thank you.

Discussion
17. The authors provide a balanced interpretation of the results, including its generalisability. In terms of limitations it would be useful to comment on the potential direction and magnitude of the potential bias that could result from high rates of drop-outs identified in the study limitations
Good point. We have added a comment on Exclusions at the start of Results and addressed potential bias in the Limitations.

“However, over 1000 children attended without an adult caregiver and could therefore not give consent. This may have introduced selection bias with under-representation of older and less severely unwell children (who may be more likely to attend without an adult caregiver).”
